# Sclerotherapy with absolute alcohol in a child with spinal aneurysmal bone cyst: A case report

**DOI:** 10.1016/j.radcr.2024.07.054

**Published:** 2024-08-13

**Authors:** Eppy Buchori Aristiyadi K, Abdul Kadir Hadar, Hermin Aminah Usman, Andreas Klemens Wienanda, Harry Galuh Nugraha, Hilman

**Affiliations:** a Department of Radiology, Faculty of Medicine Padjadjaran University, Dr. Hasan Sadikin Hospital Bandung, West Java, Indonesia; b Department of Orthopedic, Faculty of Medicine Padjadjaran University. Dr. Hasan Sadikin Hospital Bandung, West Java, Indonesia; c Department of Pathology Anatomy, Faculty of Medicine Padjadjaran University, Dr. Hasan Sadikin Hospital Bandung, West Java, Indonesia

**Keywords:** Sclerotherapy, Absolute alcohol, Aneurysmal bone cyst, Case report

## Abstract

Aneurysmal bone cyst (ABC) is a benign and locally proliferative vascular disorder in the form of a non-neoplastic bone lesion commonly found in children and young adults. Several treatments and therapeutic options are available. Percutaneous sclerotherapy is an alternative treatment for ABC with less morbidity than other therapies. An 11-year-old girl presented with a lump in her left flank since 10 months ago with paresthesia, and leg weakness. The patient was unable to raise her legs and walk. The patient underwent posterior surgical and stabilization procedures with tumor extirpation. Three months postsurgery, the lump progressively increased and tenderness. MRI showed an expansile destructive lytic lesion, firm borders, regular margins, and multiple septa with clear transition zones, without periosteal reactions, forming a picture of a “soap bubble appearance” surrounding the lumbar paravertebral. The patient underwent sclerotherapy using 5 ml of absolute alcohol under visual fluoroscopy guidance. After the sclerotherapy, the patient showed clinical improvement and decreased lump size. No side effects or massive bleeding were experienced postsclerotherapy. Thoracolumbar x-ray post sclerotherapy showed a decreased mass size in the posterior lumbar area.

This case demonstrates that sclerotherapy presents a secure alternative for pediatric patients in contrast to spinal ABC surgery. It offers minimal invasiveness and reduced morbidity. The percutaneous administration of absolute alcohol proves effective in treating spinal ABC. In this case, the patient experienced clinical improvement, leading to a decrease in lump size. There were no instances of significant bleeding around the lump postsclerotherapy.

## Introduction

An Aneurysmal Bone Cyst (ABC) is a benign and proliferative vascular disorder characterized by non-neoplastic bone lesions commonly found in children and young adults. Seventy-five percent of ABC cases occur in patients under 20 years old. ABC often occurs in long bones, particularly in the eccentric metaphysis of the femur and tibia [1]. ABC in the spine is uncommon and challenging to treat due to complex neurovascular tissue structures. The tumor comprises abundant lytic fluid and is enveloped by thin bone. ABC tends to be fragile, leading to pathological bone fractures that threaten bone stability and function [2,3].

Various treatment options and therapeutic interventions are available for managing ABC. Traditionally, conventional methods such as curettage with or without bone grafting have been used [4,5]. Moreover, selective arterial embolization, radiotherapy, or a combination of these approaches are viable options [6,7]. Percutaneous sclerotherapy offers a safe alternative to surgery for ABC, with lower morbidity outcomes compared to other treatments. Resection surgery or curettage on large lesions can result in bone defects, deformities, and even functional abnormalities, especially in children [8]. Various sclerosing agents, including absolute alcohol, ethibloc, calcium sulfate, polidocanol, and N-2-butyl-cyanoacrylate, have shown promising results. Lambot et al. reported that percutaneous injection of absolute alcohol in 29 children with ABC in the extremities and trunk yielded efficient and safe results [9]. In this case report, we present our experience with percutaneous sclerotherapy using absolute alcohol for the treatment of ABC in the spine of a pediatric patient.

## Case report

**Fig. 1:**
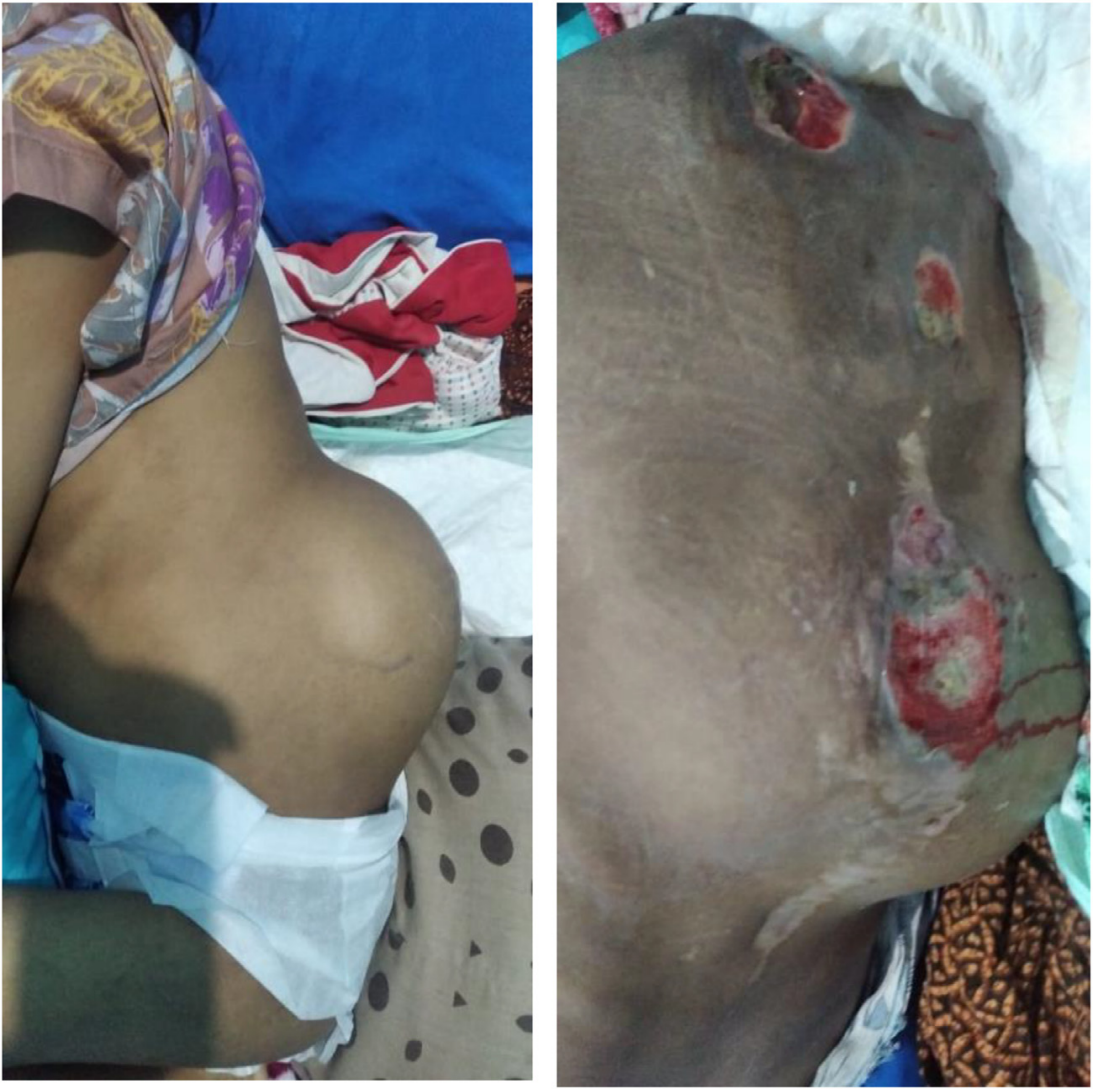
Clinical image of a lump in the left flank.

An 11-year-old girl reported a lump in her left flank that had persisted for the last 10 months. She had experienced a fall before the appearance of the lump, with her left flank contacting the ground. At that time, the lump was characterized as firm, movable, and tender upon palpation. Additionally, she expressed discomfort in the left flank region. The patient underwent surgery to remove the tumor and stabilize the posterior. However, three months after the procedure, the lump was observed to have increased in size to approximately 11 x 8 x 5 cm. It was firm to the touch, not movable, and tender upon palpation. The patient also reported experiencing pain radiating from her waist to her groin. She was encountering weakness in both legs, which hindered her ability to lift them and walk. Furthermore, she described numbness and tingling sensations in both legs. There was no history of malignancy or similar complaints in the patient's family (Fig. 1).

**Fig. 2:**
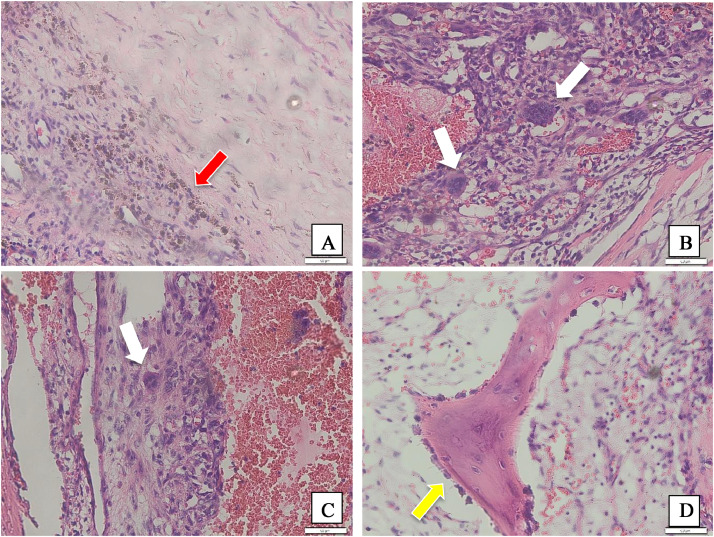
Histologic features of spinal aneurysmal bone cyst. (A) Hemosiderin pigment deposits (red arrow), magnification 200x, and (B and C) osteoclast-like giant cells (white arrow), magnification 200x, were observed. (D) Lamellar bone with normal osteocytes and osteoblastic rimming (yellow arrow) was also evident, magnification 200x.

After surgery, a sample of the lump is taken for anatomical pathology examination. During the anatomical pathology examination, the received tissue displayed a brownish-white, rubbery texture, with certain regions resembling bone density. Upon sectioning, some areas appeared solid while others were hollow, with the cavities containing blood clots. Microscopic examination of the specimen from lumbar vertebrae 2-3 revealed dense hyperplastic fibroblast cell growth within a stromal connective tissue, with nuclei within normal limits. Furthermore, there were dilated and congested blood vessels, accompanied by significant hemorrhaging. Hemosiderin pigment deposits and osteoclast-like giant cells were observed. Lamellar bone with normal osteocytes and osteoblastic rimming was also evident. Muscle tissue and mature fat cells with normal nuclei at the periphery were present within normal limits. Based on these anatomical pathology findings, the diagnosis of an aneurysmal bone cyst in the region of lumbar vertebrae 2-3 was established (Fig. 2).

Before the first surgery, the patient underwent x-ray and ultrasound examination. The x-ray showed a soft tissue mass without calcification extending from lumbar vertebrae 3-5 in the posterior lumbar region. A destructive fracture of the body of lumbar vertebra 3, caused by the mass, was evident. A lytic-destructive lesion was also present in the left femoral head, accompanied by tissue swelling and widening of the acetabular joint space. There was a kyphotic curve of the lumbar vertebrae, centered at lumbar vertebra 3, with Cobb's angle of 45 degrees. Other intervertebral discs and neural foramina appeared normal, and other pedicles were within normal limits.

**Fig. 3:**
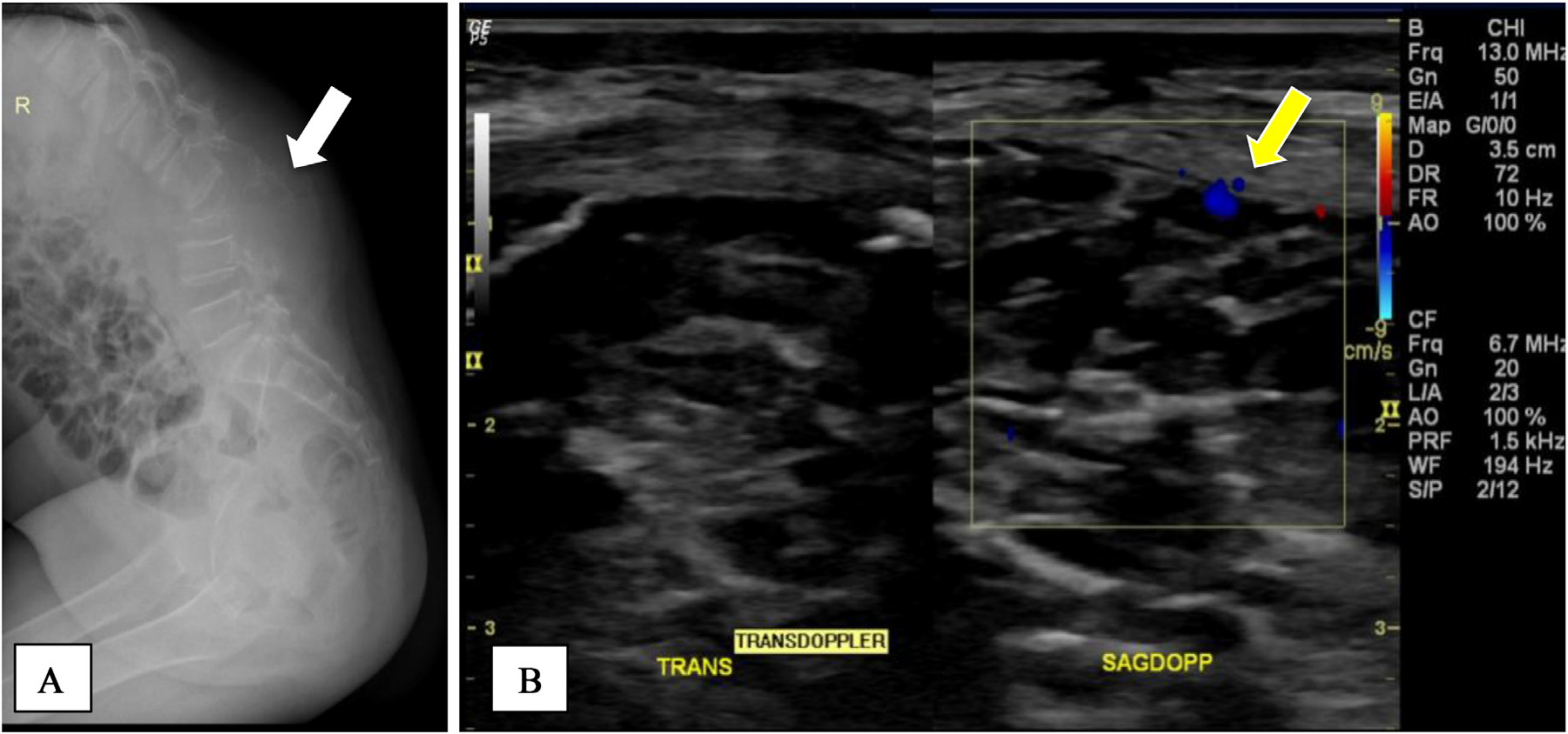
The x-ray (A) before the surgery showed a soft tissue mass without calcification extending from lumbar vertebrae 3-5 in the posterior lumbar region (white arrow). Ultrasonography with Doppler color (B) showed intramass vascularity (yellow arrow).

**Fig. 4:**
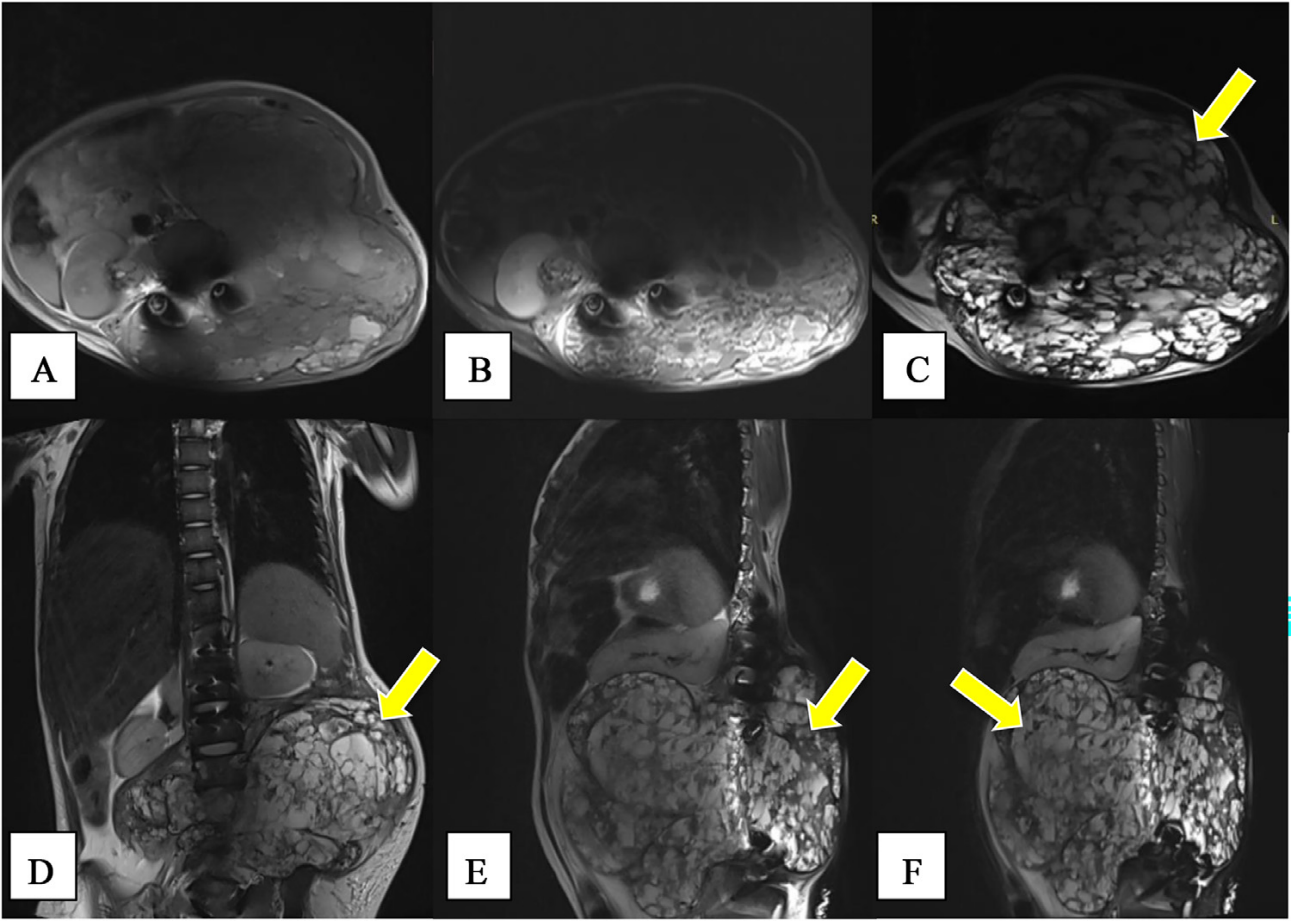
Recurrence after first surgical Magnetic Resonance Imaging (MRI). MRI the lumbosacral region, including T1WI axial noncontrast (A), T1WI axial contrast (B), T2WI axial (C), T2WI coronal (D), T2WI sagittal (E), and T2 Dixon W sagittal (F) showed a sharp transition zone without periosteal reaction formed a “soap bubble appearance” (yellow arrow) surrounding the lumbar paravertebral area.

An inhomogeneous hypoechoic mass with ill-defined borders, irregular edges, and calcifications was identified during the ultrasonography examination. The lump measured around 10.28 cm deep in the left flank region. Numerous thick-walled anechoic lesions were present within the mass. Color Doppler examination revealed intramass vascularity (Figs. 3 and 4).

The patient underwent an MRI scan of the lumbosacral region due to recurrence postsurgery. MRI scan of the lumbosacral region, including axial-sagittal TI WI, axial-sagittal-coronal T2WI, and sagittal T2 fat suppression sequences. Both contrast-enhanced and noncontrast MRI scans were conducted. The MRI images depicted an expansive, lytic, and destructive mass with clearly delineated borders, regular edges, and multiple septations, measuring approximately 18.64 x 20.80 x 16.24 cm. A sharp transition zone without periosteal reaction formed a “soap bubble appearance” (yellow arrow) surrounding the lumbar paravertebral area. The mass appeared to obliterate the longissimus, iliocostalis, internal abdominal oblique, quadratus lumborum, anterior longitudinal ligament, posterior longitudinal ligament, ligamentum flavum, interspinous ligament and obstructed the spinal canal at that level. It exhibited homogeneously hypointense signal intensity on T1WI and homogeneously hyperintense signal intensity on T2WI and T2 Dixon. Postcontrast scanning revealed heterogeneous enhancement. These observations are consistent with a diagnosis of an aneurysmal bone cyst.

**Fig. 5:**
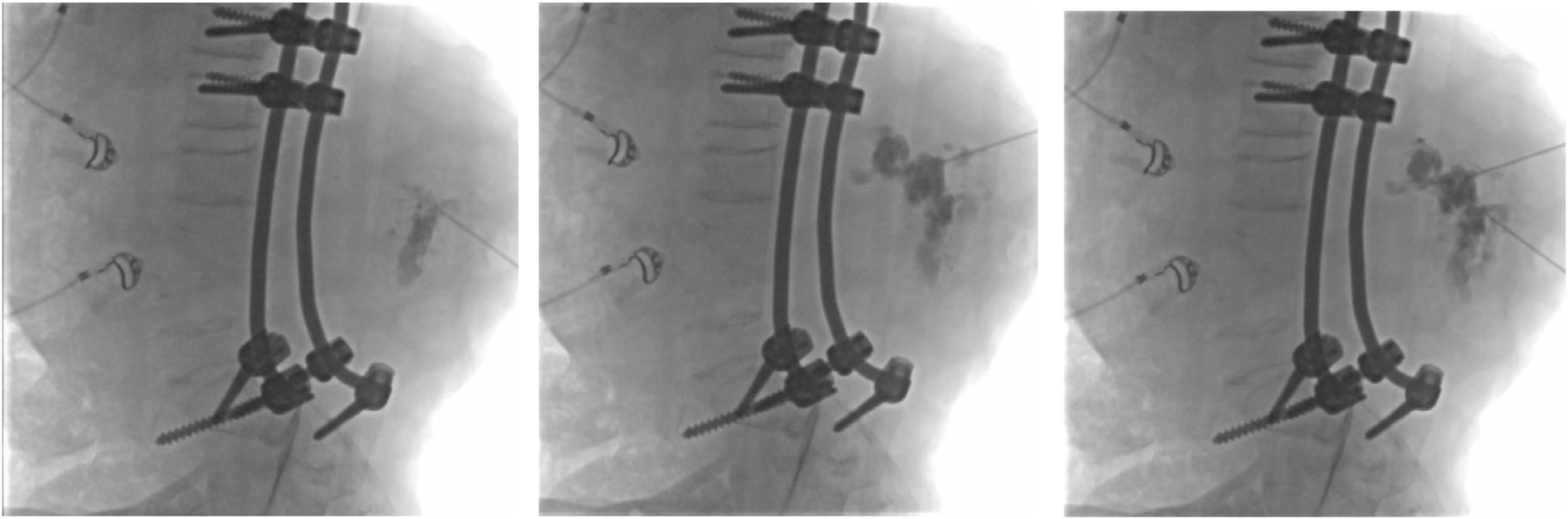
Percutaneous sclerotherapy with 5 ml absolute alcohol for an aneurysmal bone cyst.

During MRI examination, scoliosis was evident, with centralization observed at the body of lumbar vertebra 3, along with a kyphotic curvature and posterior angulation also centered at this level. Additionally, deformity and destruction of the bodies of lumbar vertebrae 2-4 were observed, resulting in homogeneously hypointense signal intensity on T1WI and homogeneously hyperintense signal intensity on T2WI and T2 Dixon sequences. Postcontrast scanning revealed diverse enhancement patterns. The bodies of the remaining thoracolumbar vertebrae exhibited normal shape and signal intensity. The interspinous space was within the expected parameters. Other intervertebral discs and articular fascia of the lumbosacral vertebrae appeared normal. Signal intensity of the nucleus pulposus and annulus fibrosus components in other intervertebral discs was normal. The spinal canal, spinal cord, and filum terminale within the scanned area displayed normal shape and signal intensity. The conus medullaris was located at the level of the body of lumbar vertebra 1. Signal intensity at the facet joints was normal. Posterior stabilization was observed from thoracic 11 to the sacral region in the MRI image (Fig. 5).

The patient received percutaneous sclerotherapy for an aneurysmal bone cyst in vertebrae lumbalis area. Before the procedure, the left flank area was prepared with aseptic and antiseptic measures. Local anesthesia was applied at the site of the puncture. Under fluoroscopic guidance, the lesion site was punctured. Contrast material was then injected at the puncture site to fill the cyst. Subsequently, sclerotherapy was administered using 5 ml of absolute alcohol through the puncture needle. The patient was regularly monitored and evaluated in the inpatient ward following the procedure.

**Fig. 6:**
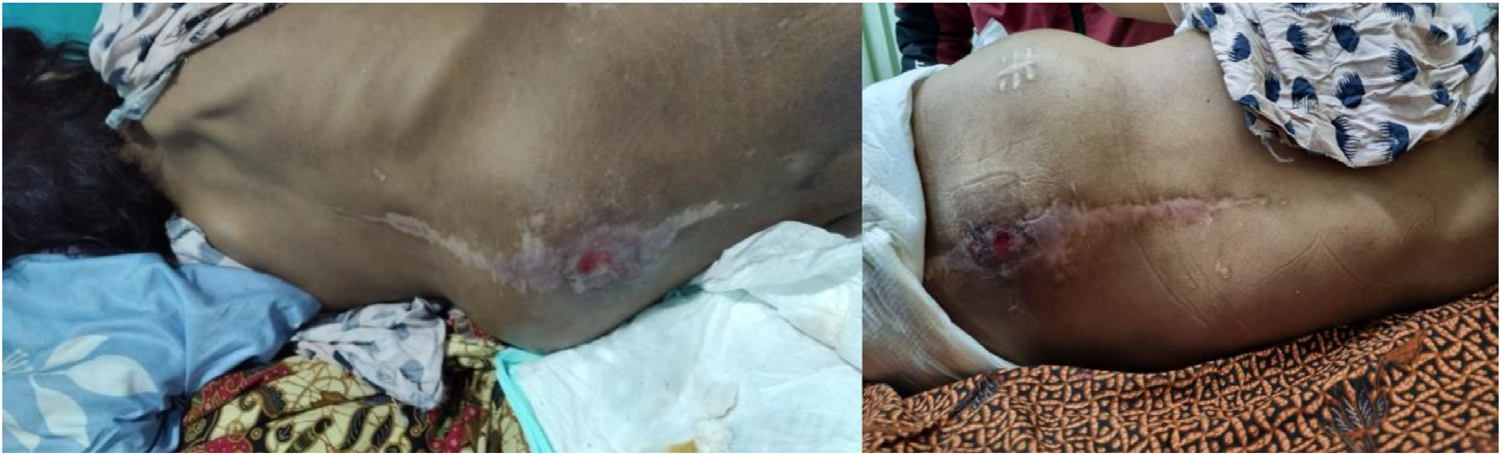
Clinical image post percutaneous sclerotherapy.

**Fig. 7:**
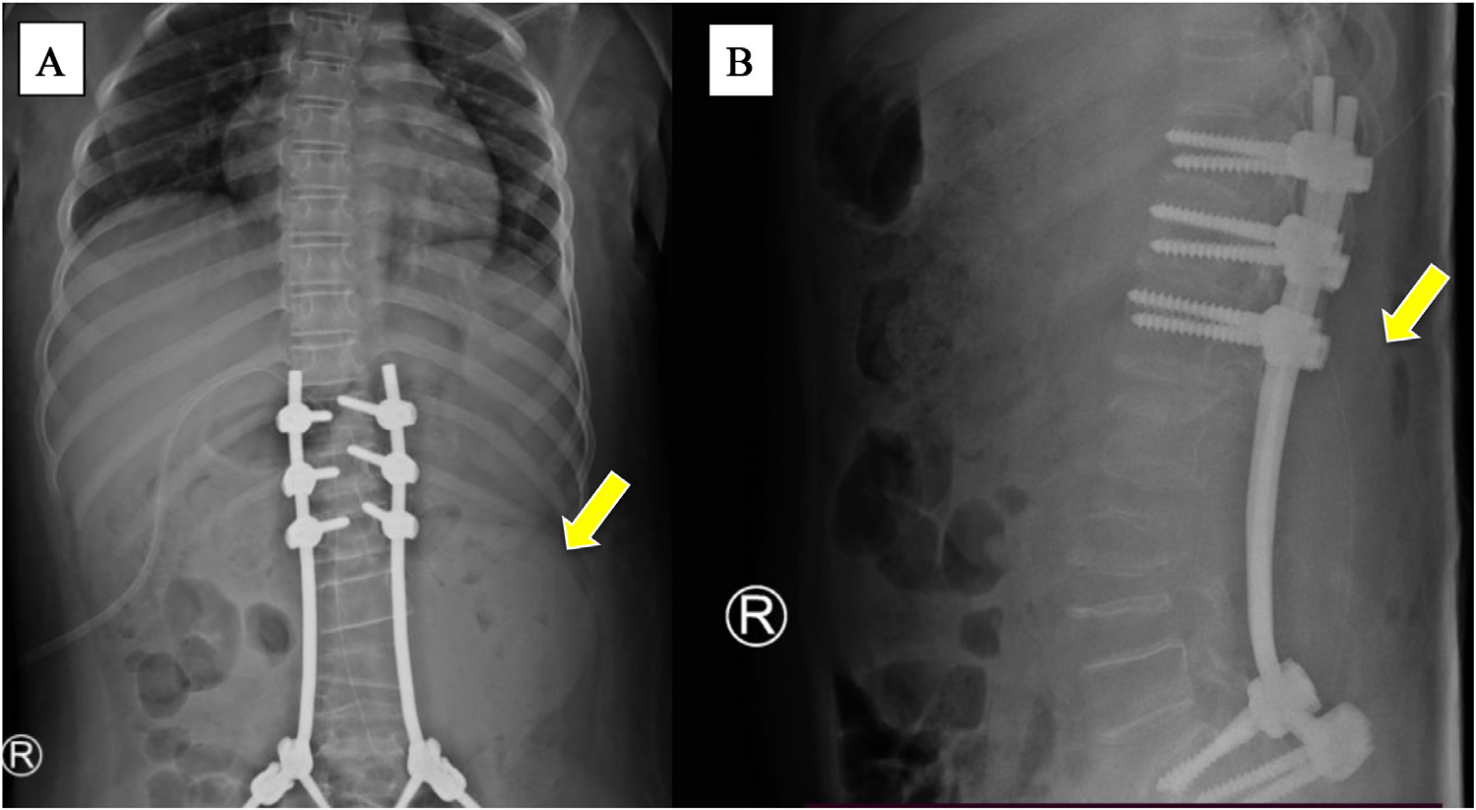
Post percutaneous sclerotherapy x-ray: AP view (A), and lateral view (B) showed the mass size was reduced.

After the sclerotherapy procedure, the patient underwent thorough monitoring, and no complications or deterioration of the patient's condition were observed (Figs. 6 and 7).

After the x-ray examination postprocedure, the mass size in the lumbar posterior region was reduced (yellow arrow). Nonetheless, the mass has not entirely vanished, indicating the need for more intensive intervention to attain improved outcomes.

## Discussion

An Aneurysmal Bone Cyst (ABC) is a benign and proliferative vascular disorder characterized by non-neoplastic bone lesions commonly found in children and young adults. ABC often occurs in long bones, particularly in the eccentric metaphysis of the femur and tibia [1]. ABCs occurring in the spine are less frequently observed. ABC tends to be fragile, leading to pathological bone fractures that threaten bone stability and function [2,3].

In this case, an 11-year-old girl reported a lump in her left flank that had persisted for the last 10 months. The lump was characterized as firm, movable, and tender upon palpation. The patient also reported experiencing pain radiating from her waist to her groin. He was encountering weakness in both legs, which hindered her ability to lift them and walk. Furthermore, she described numbness and tingling sensations in both legs. The anatomical pathology results confirmed the presence of an aneurysmal bone cyst. The x-ray images of the lumbosacral region revealed the occurrence of this mass in an uncommon location, specifically within the spine.

MRI can serve as an effective diagnostic tool for confirming the presence of an aneurysmal bone cyst. According to Peter et al. [10], MRI typically reveals characteristic features of an aneurysmal bone cyst, such as an expansile lytic lesion accompanied by septations. Fluid-to-fluid and soap bubble appearance are common MRI findings in patients with aneurysmal bone cysts. The MRI findings in this patient align with those described in the literature, showing an expansile destructive lytic mass with well-defined borders, regular edges, multiple septations, a sharp transition zone, and no periosteal reaction, forming a soap bubble appearance surrounding the lumbar paravertebral area. The mass obstructs the spinal canal at that level, resulting in homogeneously hypointense signal intensity on T1WI and homogeneously hyperintense signal intensity on T2WI and T2 Dixon sequences. Postcontrast scanning revealed heterogeneous enhancement, further supporting the diagnosis of an aneurysmal bone cyst. As reported in several studies, ABCs can lead to pathological bone fractures that compromise bone stability and function [2,3]. In this patient's MRI, deformity and destruction of lumbar vertebral bodies 2-4 were evident, contributing to instability.

Percutaneous sclerotherapy is viewed as a safer alternative to surgery for ABC due to its lower morbidity rate. Resection surgery or curettage on large lesions can result in bone defects, deformities, and even functional abnormalities, especially in children [8]. Hence, in this case report, the patient underwent percutaneous sclerotherapy using a sclerosing agent as an elective treatment for ABC. Various sclerosing agents are available for percutaneous sclerotherapy and have shown favorable outcomes [9]. Literature suggests a dosage of 0.2 ml/kg of body weight of absolute alcohol to achieve optimal sclerotherapy results with minimal risks [9]. In this instance, the patient received a 5 ml dose of absolute alcohol during sclerotherapy for the aneurysmal bone cyst.

Several studies have indicated that younger patients frequently encounter adverse effects and diminished responses to sclerotherapy treatments [[11], [12], [13]]. In this case report, the patient showed clinical improvement, with a reduction in the lump size. The lump was observed to have decreased in size to approximately 10 x 6 x 4 cm. The patient did not report any side effects after undergoing sclerotherapy. There was no evidence of significant bleeding in the vicinity of the lump. Guilbaud, et al. outlined potential complications associated with sclerotherapy performed under general anesthesia, including pulmonary embolism, osteomyelitis, and aseptic osteitis [14]. According to Josee et al., handling ABCs in pediatric patients' brain and spinal cord requires careful consideration due to potential risks. One of these risks includes the potential leakage of sclerosing agents, which may penetrate the central nervous system and induce damage [15].

## Conclusion

This case demonstrates that sclerotherapy presents a secure alternative for pediatric patients in contrast to spinal ABC surgery. It offers minimal invasiveness and reduced morbidity. The percutaneous administration of absolute alcohol proves effective in treating spinal ABC. In this case, the patient experienced clinical improvement, leading to a decrease in lump size. There were no instances of significant bleeding around the lump postsclerotherapy.

## Patient consent

Written informed consent for publication of their case was obtained from our patient.

## Author Contributions

All the author contribution to Conceptualization, Validation, Writing Original Draft, Writing Review and Editing, Visualization, and Supervision.
